# Pharmacological Blockade of Soluble Epoxide Hydrolase Attenuates the Progression of Congestive Heart Failure Combined With Chronic Kidney Disease: Insights From Studies With Fawn-Hooded Hypertensive Rats

**DOI:** 10.3389/fphar.2019.00018

**Published:** 2019-01-23

**Authors:** Šárka Vacková, Libor Kopkan, Soňa Kikerlová, Zuzana Husková, Janusz Sadowski, Elzbieta Kompanowska-Jezierska, Bruce D. Hammock, John D. Imig, Miloš Táborský, Vojtěch Melenovský, Luděk Červenka

**Affiliations:** ^1^Center for Experimental Medicine, Institute for Clinical and Experimental Medicine, Prague, Czechia; ^2^Department of Physiology, Faculty of Science, Charles University, Prague, Czechia; ^3^Department of Renal and Body Fluid Physiology, Mossakowski Medical Research Centre, Polish Academy of Sciences, Warsaw, Poland; ^4^Department of Entomology, UCD Cancer Center, University of California, Davis, Davis, CA, United States; ^5^Department of Pharmacology and Toxicology, Medical College of Wisconsin, Milwaukee, WI, United States; ^6^Department of Internal Medicine I, Cardiology, University Hospital Olomouc, Palacký University, Olomouc, Czechia; ^7^Department of Cardiology, Institute for Clinical and Experimental Medicine, Prague, Czechia; ^8^Department of Pathophysiology, Second Faculty of Medicine, Charles University, Prague, Czechia

**Keywords:** congestive heart failure, chronic kidney disease, soluble epoxide hydrolase inhibitor, hypertension, renin-angiotensin-aldosterone system

## Abstract

An association between congestive heart failure (CHF) and chronic kidney disease (CKD) results in extremely poor patient survival rates. Previous studies have shown that increasing kidney epoxyeicosatrienoic acids (EETs) by blocking soluble epoxide hydrolase (sEH), an enzyme responsible for EETs degradation, improves the survival rate in CHF induced by aorto-caval fistula (ACF) and attenuates CKD progression. This prompted us to examine if sEH inhibitor treatment would improve the outcome if both experimental conditions are combined. Fawn-hooded hypertensive (FHH) rats, a genetic model showing early CKD development was employed, and CHF was induced by ACF. Treatment with an sEH inhibitor was initiated 4 weeks after ACF creation, in FHH and in fawn-hooded low-pressure (FHL) rats, a control strain without renal damage. The follow-up period was 20 weeks. We found that ACF FHH rats exhibited substantially lower survival rates (all the animals died by week 14) as compared with the 64% survival rate observed in ACF FHL rats. The former group showed pronounced albuminuria (almost 30-fold higher than in FHL) and reduced intrarenal EET concentrations. The sEH inhibitor treatment improved survival rate and distinctly reduced increases in albuminuria in ACF FHH and in ACF FHL rats, however, all the beneficial actions were more pronounced in the hypertensive strain. These data indicate that pharmacological blockade of sEH could be a novel therapeutic approach for the treatment of CHF, particularly under conditions when it is associated with CKD.

## Introduction

Congestive heart failure (CHF) presents a serious medical problem affecting about 4% of the adult population (Ziaeian and Fonarow, 2016; Benjamin et al., 2017). The incidence of chronic kidney disease (CKD) is also increasing, reaching a level of 8–16% worldwide (Jha et al., 2013; U.S. Renal Data System, 2015) which makes it a growing public health problem. It will be noticed that CHF and CKD can have deleterious effects on each other, through the activation of vicious cycles resulting ultimately in extremely poor outcomes in patients with CHF and CKD associated (Braam et al., 2014; Schefold et al., 2016; Mullens et al., 2017; Arrigo et al., 2018; House, 2018). In spite of an array of therapeutic approaches applied, the survival prognosis of patients with CHF who exhibit kidney disease remains bleak. Therefore, there is an obvious need for focused experimental studies that would examine the value of novel therapeutic approaches.

Within the research of the pathophysiological background of hypertension, CHF, and CKD, considerable attention has been focused on the role of epoxyeicosatrienoic acids (EETs), cytochrome P-450 (CYP)-dependent metabolites of arachidonic acid (AA). Indeed, increased EETs levels was reported to have important antihypertensive and organ-protective actions (Elmarakby, 2012; Kujal et al., 2014; Fan and Roman, 2017; Imig, 2018). However, direct therapeutic potential of EETs is limited because they are rapidly metabolized to biologically inactive dihydroxyeicosatrienoic acids (DHETEs) by soluble epoxide hydrolase (sEH). On the other hand, blocking sEH and increasing tissue EETs bioavailability produced antihypertensive and cardio- and renoprotective effects (Imig, 2018). Moreover, in the spontaneously hypertensive heart failure rat, an inbred, genetically homogenous model that mirrors human hypertension-associated heart failure (McCune et al., 1990), an alteration of the gene encoding sEH (*Ephx2*) facilitated CHF progression and was identified as a heart failure susceptibility gene (Monti et al., 2008).

Collectively, these findings suggest that pharmacological blockade of sEH could present a new approach for CHF treatment, particularly when CHF is combined with CKD. However, no evidence is so far available to indicate that chronic sEH inhibition results in a prolongation of life in individuals with advanced CHF associated with evident kidney disease.

In order to study the pathophysiological mechanism(s) of CHF and possible novel therapeutic measures, rats with aorto-caval fistula (ACF) in which CHF is induced by volume overload was introduced 40 years ago (Hatt et al., 1980). This model has many features similar to untreated human CHF (Brower et al., 1996; Abassi et al., 2011; Melenovsky et al., 2012, 2018; Cohen-Segev et al., 2014; Červenka et al., 2015) and is now recommended by American Heart Association for testing therapeutic strategies for CHF (Houser et al., 2012). In addition, fawn-hooded hypertensive rats (FHH) represent a unique genetic model of spontaneous hypertension characterized by early development of kidney disease (Proovoost, 1994; Doleželová et al., 2016). Thus, FHH seems to be an optimal experimental model to study the course of combined CHF and CKD.

Making use of suitable experimental models, we have undertaken to evaluate the effects of sEH inhibitor treatment on morbidity and mortality in male FHH with ACF-induced CHF. To gain a more detailed insight in the possible mechanism(s) underlying the expected beneficial action of chronic sEH inhibition, we determined concentrations of CYP-derived AA metabolites. Given the established role of the renin-angiotensin system (RAS) promoting the progression of CHF (Braunwald, 2015; Ziaeian and Fonarow, 2016; Packer and McMurray, 2017 CEPP), urinary angiotensinogen excretion and concentrations of angiotensin II (ANG II) were also determined. All these indices were compared between sham-operated FHH and either untreated or treated ACF FHH on one side and their sham-operated and either untreated or treated ACF normotensive counterparts [fawn-hooded low-pressure (FHL) rats] on the other side, the latter strain reportedly resistant to renal damage (Proovoost, 1994; Doleželová et al., 2016). Finally, to further elucidate possible mechanism(s) underlying the beneficial effects of the treatment with sEH inhibitor on the course of ACF-induced CHF, we assessed the cardiac structure and function, using echocardiography and invasive pressure-volume analyses of the left ventricle (LV). This was done after 2-weeks of treatment, because at this stage (i.e., 6 weeks after induction of ACF) untreated ACF FHH still exhibited 100% survival rate. On the other hand, this was just 1 week before the rats began to die and it was particularly interesting to find out if the treatment regime applied at this stage would beneficially influence cardiac function.

## Materials and Methods

### General Methodological Procedures

#### Ethical Approval and Animals

The studies followed the guidelines and practicesestablished by the Animal Care and Use Committee of the Institute for Clinical and Experimental Medicine, which accord with the national law and with American Physiological Society guiding principles for the care and use of vertebrate animals in research and training, and were approved by the Animal Care and Use Committee of the Institute for Clinical and Experimental Medicine and, consequently, by the Ministry of Health of the Czechia (project decision 17124/2016-OZV-30.0.8.3.16/2).

Male FHH and FHL rats, at the initial age of 12 weeks, derived from several litters, were randomly assigned to experimental groups. In order to obtain reliable data regarding the effects of the treatment regimen on the survival rate, high initial *n*-values were employed (not so for sham-operated animals) to enable valid comparison of the long-term survival rate.

#### CHF Model, Pharmacological Therapeutic Regimen and General Methodological Procedures

Rats were anesthetized (tiletamine + zolazepam, Virbac SA, Carros Cedex, France, 8 mg/kg; and xylasine, Spofa, Czechia, 4 mg/kg intramuscularly) and CHF was induced by volume overload caused by ACF created using needle technique as employed and validated by many investigators including our own group (Oliver-Dussault et al., 2010; Abassi et al., 2011; Melenovsky et al., 2012, 2018; Cohen-Segev et al., 2014; Brower et al., 2015; Červenka et al., 2015, 2016; Kala et al., 2018). Briefly, after exposure of the abdominal aorta and inferior vena cava between the renal arteries and iliac bifurcation, the aorta was temporarily occluded at this segment for about 40 s. An 18-gauge needle (diameter 1.2 mm) was inserted into the abdominal aorta and advanced across its wall into the inferior vena cava to create ACF. Thereafter the needle was withdrawn and the puncture site was sealed with cyanoacrylate tissue glue. Successful creation of ACF was confirmed by inspection of pulsatile flow of oxygenated blood from the abdominal aorta into the vena cava. Sham-operated rats underwent an identical procedure but without creating ACF. To inhibit sEH, *cis*-4-[4-(3-adamantan-1-yl-ureido) cyclohexyloxy]benzoic acid (*c*-AUCB) was used, which was prepared freshly and given in drinking water at 3 mg/L. The appropriate amount of *c*-AUCB was dissolved under gentle warming in polyethylene glycol and added with rapid stirring to warm drinking water to obtain 0.1% aqueous solution of polyethylene glycol. The dose of *c*-AUCB was selected based on our recent studies where it elicited substantial increases in tissue concentration of EETs without altering RAS activity (Sporková et al., 2014; Červenka et al., 2015; Kala et al., 2018).

Rat total angiotensinogen concentrations were measured in urine samples by a solid phase sandwich Enzyme-linked Immunosorbent Assay, using the commercially available ELISA kit (JP27414, IBL Int., Hamburg, Germany) as described in our recent study (Čertíková Chábová et al., 2018).

The samples for measurement of plasma and kidney ANG II concentrations were obtained from conscious decapitated rats because it is established that they are higher than those measured under anesthesia (Husková et al., 2006a,b). ANG II concentrations were assessed by radioimmunoassay based on the original procedure developed by Fox et al. (1992) and further modified and validated in our laboratory (Husková et al., 2016). This method is described in detail in our recent publication (Čertíková Chábová et al., 2018).

The levels of arachidonic acid metabolites: EETs (specifically: 5,6-EET, 8,9-EET, 11,12-EET, and 14,15-EET) and DHETEs (the biologically active and inactive, respectively, products of CYP epoxygenase pathway) were measured in the kidney cortex and the LV myocardium. The EETs/DHETEs ratio was calculated from total concentrations of EETs and of DHETEs. In the same cortex samples the levels of hydroxyeicosatetraenoic acids (HETEs), the products of CYP ω-hydroxylase pathway were determined, specifically 5-HETE, 8-HETE, 11-HETE, 12-HETE, 15-HETE, 19-HETE, and 20-HETE, which were separately determined and pooled for presentation: these metabolites are biologically the most active products of the CYP epoxygenase and hydroxylase pathways (Jamieson et al., 2017; Imig, 2018). For these analyses, 20–40 mg of tissue was used. Homogenized tissue samples were subjected to alkaline hydrolysis and solid-phase extraction was performed as described by Rivera et al. (2004). Thereafter the samples were analyzed using high performance liquid chromatography (using Agilent 1200SL with tandem mass spectroscopy (MS) and Agilent 6460 for quantification) as described in detail previously (Alánová et al., 2015; Jíchová et al., 2016; Čertíková Chábová et al., 2018).

### Detailed Experimental Design

#### Series 1: Comparison of the Survival Rate, Albuminuria and Urinary Angiotensinogen Excretion Between ACF FHH and ACF FHL Rats and Effects of the Treatment With sEH Inhibitor

Animals underwent either sham-operation or ACF creation as described above (at the week labeled -4) and were left without treatment for 4 weeks. Previous studies have shown that after this time CHF features are fully developed but the animals are still in the compensation phase (Oliver-Dussault et al., 2010; Melenovsky et al., 2012, 2018; Červenka et al., 2015, 2016; Kala et al., 2018). At this time point (week 0) the rats were divided into the following experimental groups:

1. Sham-operated FHL + placebo (initial *n* = 10).

2. ACF FHL + placebo (initial *n* = 26).

3. ACF FHL + sEH inhibitor (initial *n* = 26).

4. Sham-operated FHH + placebo (initial *n* = 10).

5. ACF FHH + placebo (initial *n* = 29).

6. ACF FHH + sEH inhibitor (initial *n* = 27).

The follow-up period was 20 weeks (until week +20). In the weeks labeled -5 (i.e., 1 week before ACF creation), +4, +6, +8, +10, +20 after appropriate habituation training the animals were placed in individual metabolic cages and their 24-h urine was collected for determination of albuminuria and urinary angiotensinogen excretion.

#### Series 2: Effects of 10-Week Treatment With sEH Inhibitor on ANG II, EETs, DHETEs, and HETEs Concentrations and on Organ Weights

Animals were prepared as described in series 1 and in the week 0 the pharmacological treatment was initiated for a period of 10 weeks in the same experimental groups as described in series 1. At the end of experiment (in the week +10) the rats were killed by decapitation and plasma ANG II, kidney, and heart LV concentrations of ANG II, EETs, DHETEs, and HETEs were measured as described above (*n* = 10 in each experimental group). The aim of this series was to evaluate the degree of RAS activation and the activity of CYP-dependent epoxygenase and hydroxylase pathways and the effects of sEH inhibitor treatment.

#### Series 3: Effects of 2-Week Treatment With sEH Inhibitor on Basal Cardiac Function Parameters Assessed by Echocardiography and by Pressure-Volume Analysis

Animals were prepared as described in series 1 and in the week 0 the pharmacological treatment was applied for 2 weeks. At this time point (week +2) experiments were performed in the following groups:

1. Sham-operated FHL + placebo (water).

2. Sham-operated FHL + sEH inhibitor.

3. ACF FHL + placebo.

4. ACF FHL + sEH inhibitor.

5. Sham-operated FHH + placebo.

6. Sham-operated FHH + sEH inhibitor.

7. ACF FHH + placebo.

8. ACF FHH + sEH inhibitor.

(*n* = 6 in each group). At the end of the experimental protocol, animals were anesthetized by intraperitoneal (i.p.,) ketamine/midazolam combination (50 mg and 5 mg/kg of body weight, respectively) and echocardiography was performed as described in our earlier studies (Benes et al., 2011; Červenka et al., 2015). Subsequently, the rats were intubated with an appropriate cannula, relaxed with pancuronium (Pavulon, 0.16 mg/kg) and artificially ventilated (rodent ventilator Ugo Basile, Italy, FiO2 = 21%). LV function was invasively assessed using a 2F Pressure-Volume (P-V) micromanometry catheter (Millar Instruments) introduced into the LV cavity via the right carotid artery after previous vagal blockade (atropine 0.10 mg/kg), as described previously (Benes et al., 2011; Červenka et al., 2015). The volume signal was calibrated by determining end-diastolic and end-systolic volume by echocardiography, shortly before invasive recordings. The data were acquired using an 8-channel Power Lab recorder and were analyzed by Labchart Pro software (ADInstruments, Australia). The aim of this series was to evaluate the degree of the impairment of cardiac function at the stage when untreated ACF FHH began to die, and to examine the effects of treatment on cardiac function.

### Statistical Analysis

Statistical analysis of the data was performed using Graph-Pad Prism software (Graph Pad Software, San Diego, CA, United States). Comparison of survival curves was performed by log-rank (Mantel-Cox) test followed by Gehan-Breslow-Wilcoxon test. Statistical comparison of other results was made by Student’s *t*-test, Wilcoxon’s signed-rank test for unpaired data or one-way ANOVA when appropriate. The values are expressed as mean ± SEM. *P*-values below 0.05 were considered statistically significant.

## Results

### Series 1: Comparison of the Survival Rate, Albuminuria and Urinary Angiotensinogen Excretion in ACF FHH and ACF FHL Rats and Effects of the Treatment With sEH Inhibitor

**FIGURE 1:**
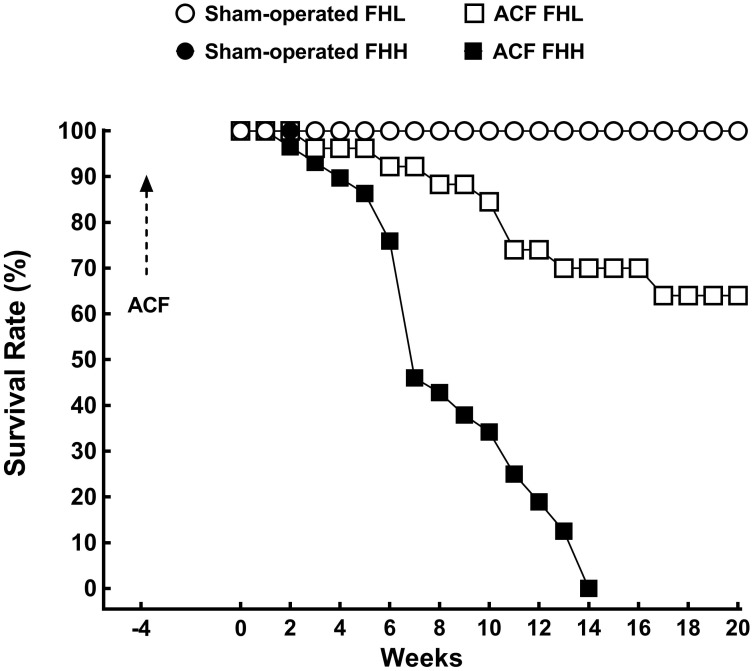
Survival rates in sham-operated fawn-hooded low-pressure (FHL), sham-operated hypertensive (FHH) rats and in untreated FHL, and FHH rats with aorto-caval fistula (ACF). The survival profiles (straight line) for sham-operated FHL and FHH were superimposable and are depicted by one symbol only (blank circles). The survival rate of ACF FHL was significantly lower than in sham-operated FHL rats and in ACF FHH it was the lowest of all experimental groups (analyzed by log-rank Mantel-Cox test followed by Gehan-Breslow-Wilcoxon test).

**FIGURE 2:**
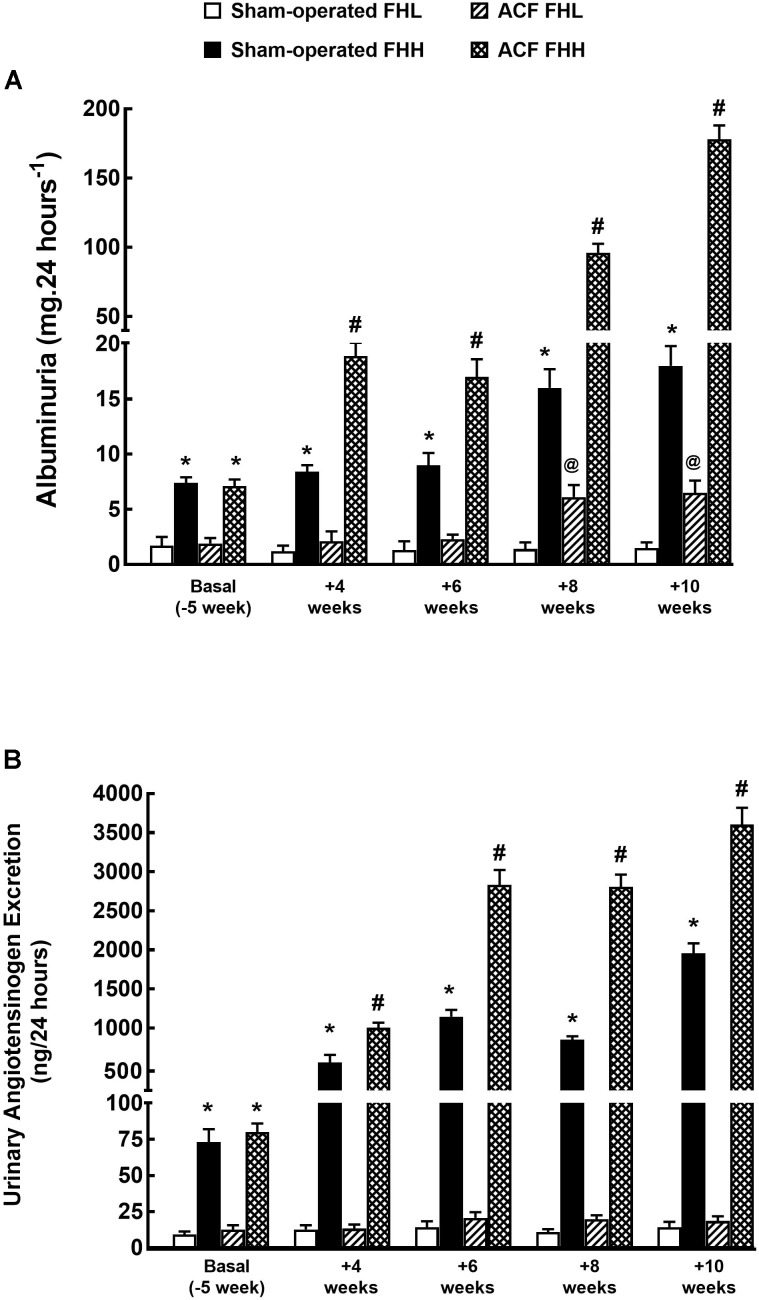
Albuminuria **(A)** and urinary angiotensinogen excretion **(B)** in sham-operated FHL, FHH rats and in FHL and FHH rats with ACF. ^∗^*P* < 0.05 for sham-operated FHH rats compared with sham-operated FHL rats at the same time point. ^#^*P* < 0.05 for ACF FFH rats compared with sham-operated FHH rats at the same time point. ^@^*P* < 0.05 ACF FHL for rats compared with sham-operated FHL rats at the same time point.

Figures 1, 2 present the data on survival rate, albuminuria and urinary angiotensinogen excretion in untreated experimental animals.

All sham-operated FHH and FHL rats survived until the end of the experiment (Figure 1). ACF FHH rats began to die at week 2 (i.e., 6 weeks after ACF creation) and all the animals died by week 14. In contrast, untreated ACF FHL rats showed higher survival rate throughout the study and the final rate at week 20 (i.e., 24 weeks after creation of ACF) was 64%.

Figure 2A shows that at the start the sham-operated FHH rats showed albuminuria that was about three-fold higher than in sham-operated FHL rats. In FHL rats it remained stable throughout the study whereas it progressively increased in sham-operated FHH: in the end it was 12-fold higher than in sham-operated FHL. Albuminuria was gradually but only slightly increasing during the study in ACF FHL rats but was still significantly lower than in sham-operated FHH rats. By contrast, in ACF FHH rats the albuminuria exhibited a progressive increase which was much steeper than in sham-operated FHH rats: at week 10 (i.e., 14 weeks after creation of ACF) it was 10-fold higher, despite the fact that the animals with the most pronounced albuminuria died early and have not been included in the final calculation.

As shown in Figure 2B, at the start (i.e., before either sham-operation or creation of ACF) the urinary angiotensinogen excretion was about seven-fold higher in sham-operated FHH rats than in the corresponding FHL group. Thereafter angiotensinogen showed a marked progressive increase throughout the experiment and at the end it was already 140-fold higher. Untreated ACF FHL rats displayed significant increases in the urinary angiotensinogen excretion, unlike the stable levels observed in the sham-operated FHL group. In untreated ACF FHH rats urinary angiotensinogen excretion dramatically increased, significantly more than in the corresponding sham-operated group: at the end it was about 90-fold higher than in untreated ACF FHL rats.

**FIGURE 3:**
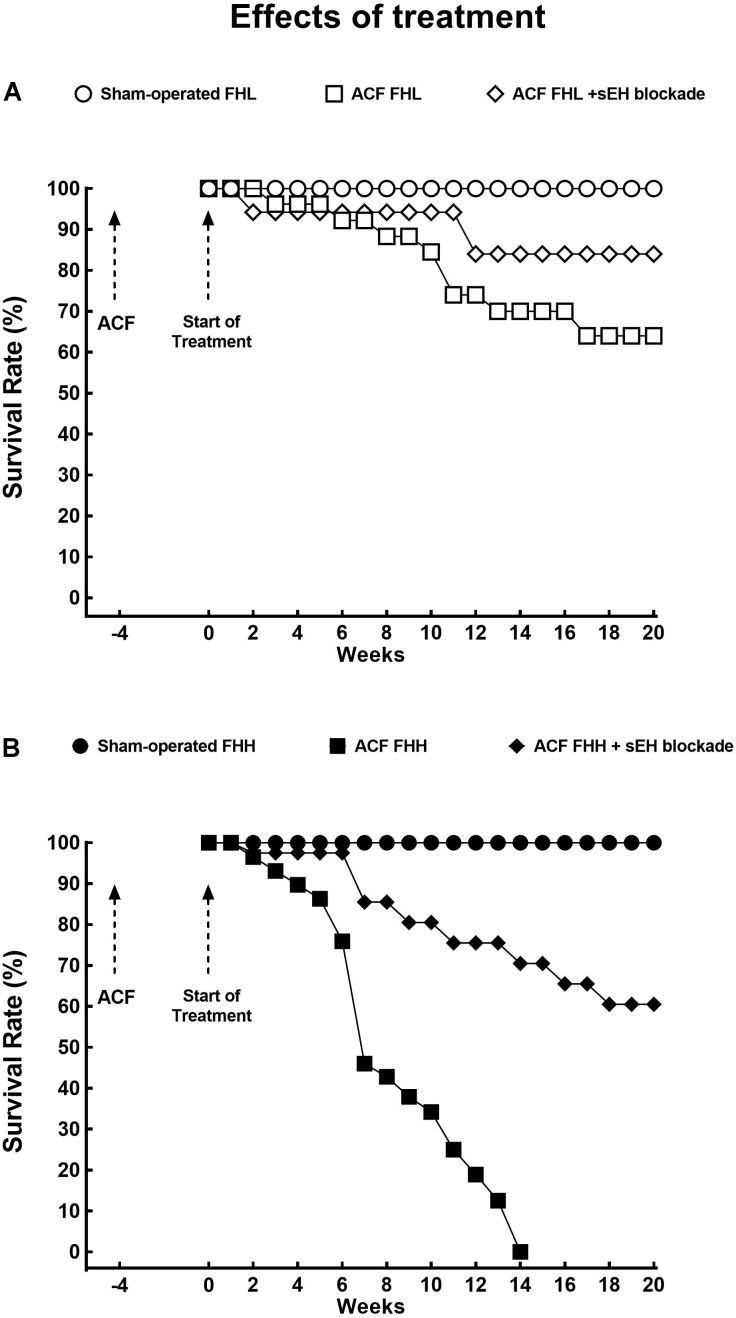
**(A)** Survival rates in sham-operated fawn-hooded low-pressure rats (FHL) with ACF and ACF FHL rats treated with a soluble epoxide hydrolase (sEH) inhibitor. **(B)** Survival rates in sham-operated fawn-hooded hypertensive (FHH) rats, untreated ACF FHH, and ACF FHH treated with inhibitor of sEH. The comparison of the survival rates curves was performed by log-rank Mantel-Cox test followed by Gehan-Breslow-Wilcoxon test.

As shown in Figure 3, sEH inhibitor treatment improved the survival rate in ACF FHL rats as well as in ACF FHH rats, but the improvement was much more pronounced in the latter group: in ACF FHL the final (week 20) survival rate was 61% whereas untreated ACF FHH rats did not survive beyond week 14 (Figure 3B).

**FIGURE 4:**
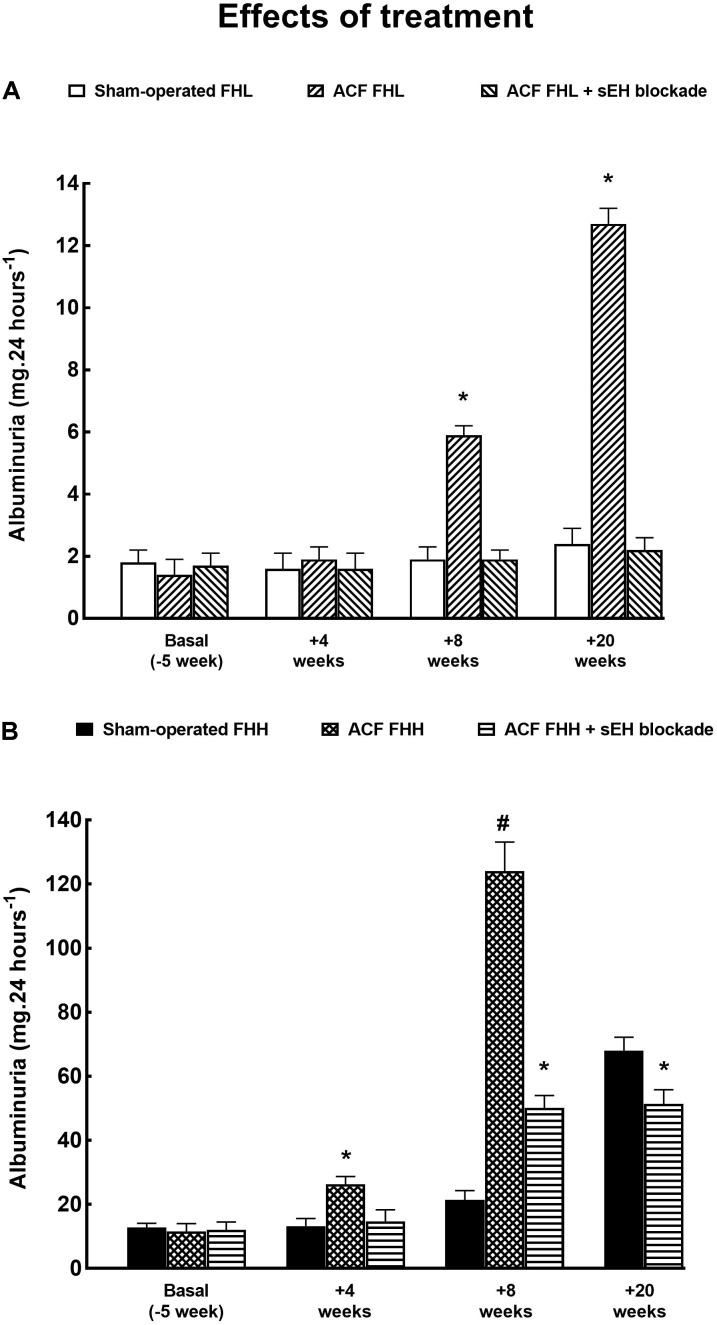
**(A)** Albuminuria in sham-operated FHL with ACF and in ACF FHL rats treated with a sEH inhibitor. **(B)** Albuminuria in sham-operated fawn-hooded hypertensive (FHH) rats, untreated ACF FHH, and ACF FHH treated with sEH inhibitor. ^∗^*P* < 0.05 compared with sham-operated counterparts at the same time point. ^#^*P* < 0.05 ACF FHH rats compared with ACF FHH rats treated with sEH inhibitor (at the same time point).

Figure 4 presents the data on the effects of sEH inhibition on the development of albuminuria in ACF animals: evidently, the long-term treatment eliminated increases in albuminuria after ACF creation in FHL rats. In the FHH group the therapeutic effect was somewhat less pronounced but at the end of the study albuminuria was not different from that observed in sham-operated animals.

**FIGURE 5:**
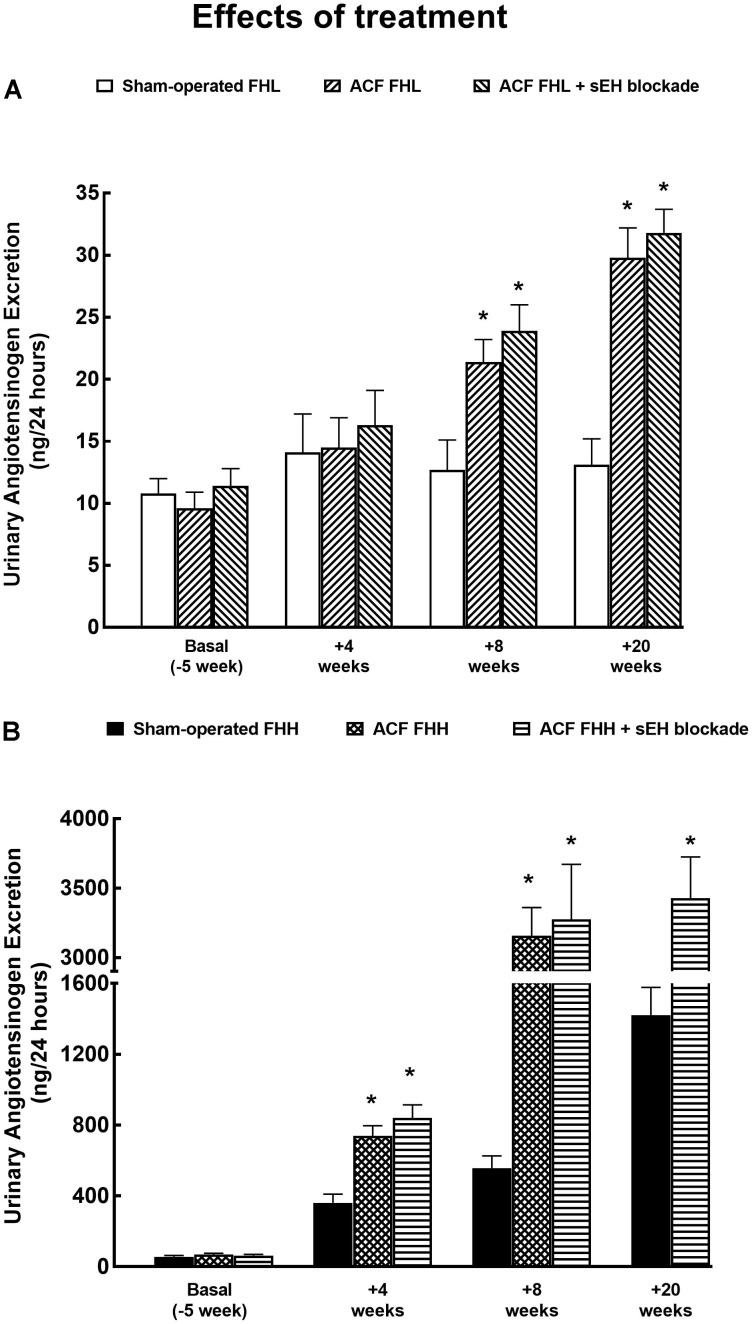
**(A)** Urinary angiotensinogen excretion in sham-operated fawn-hooded low-pressure rats (FHL) with ACF and ACF FHL rats treated with a sEH inhibitor. **(B)** Urinary angiotensinogen excretion in sham-operated fawn-hooded hypertensive (FHH) rats, untreated and treated with sEH inhibitor. ^∗^*P* < 0.05 compared with sham-operated counterparts at the same time point.

As shown in Figure 5, urinary angiotensinogen excretion increased progressively and significantly during the study in ACF FHH but not in ACF FHL rats. The treatment with sEH inhibitor did not alter the course of urinary angiotensinogen excretion, similarly in ACF FHL and in ACF FHH rats.

### Series 2: Effects of 10-Week Treatment With sEH Inhibitor on ANG II, EETs, DHETEs and HETEs Concentrations and on Organ Weights

**FIGURE 6:**
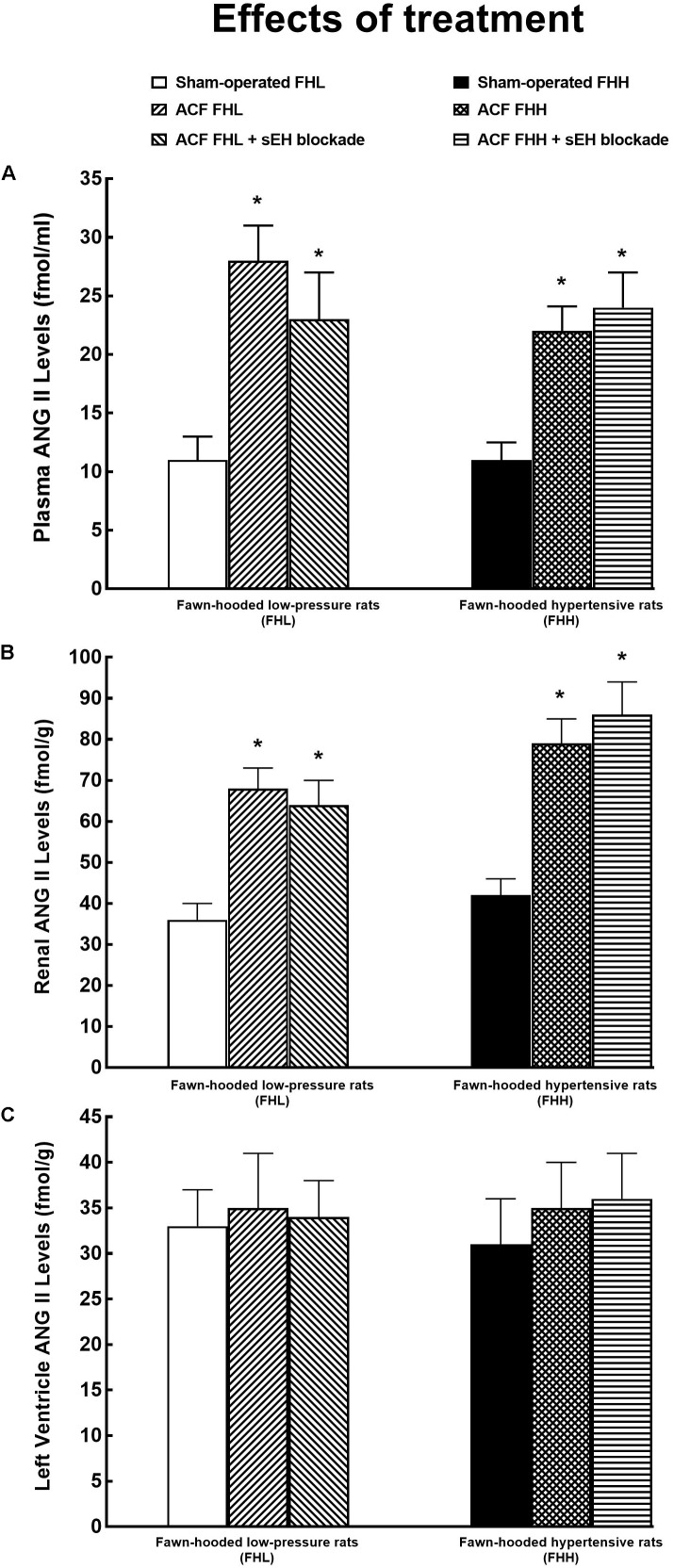
Plasma **(A)**, kidney **(B)**, and left ventricle **(C)** angiotensin II (ANG II) levels in sham-operated fawn-hooded low-pressure rats (FHL) with ACF and ACF FHL rats treated with a sEH inhibitor, and in sham-operated fawn-hooded hypertensive (FHH) rats, untreated and treated with sEH inhibitor. ^∗^*P* < 0.05 compared with the sham-operated counterparts at the same time point.

As shown in Figure 6, there were no significant differences in plasma, kidney, and LV concentrations of ANG II between sham-operated FHL and sham-operated FHH rats. The levels of plasma and kidney ANG II levels were significantly higher in untreated ACF FHL and ACF FHH rats than in their sham-operated counterparts. In contrast, LV concentrations of ANG II were not significantly higher in untreated ACF FHL and ACF FHH rats than in their sham-operated counterparts (Figure 6C). The treatment with sEH inhibitor did not modify plasma, kidney, and LV concentrations of ANG II, similarly in ACF FHL and ACF FHH rats.

**FIGURE 7:**
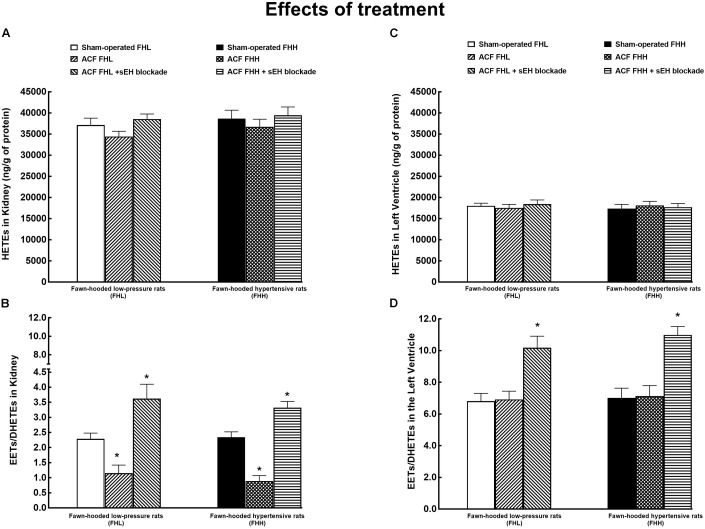
Renal tissue concentrations of hydroxyeicosatrienoic acids (HETEs) **(A)**, the renal ratio of epoxyeicosatrienoic acids (EETs) to dihydroxyeicosatrienoic acids (DHETEs) **(B)**, HETE concentrations in the left ventricle **(C)**, and EETs/DHETEs ratio in the left ventricle **(D)** in the sham-operated FHL rats with ACF and ACF FHL rats treated with the sEH inhibitor, and in sham-operated fawn-hooded hypertensive (FHH) rats, and in untreated or sEH-treated ACF FHH rats. ^∗^*P* < 0.05 compared with the sham-operated counterparts.

Figure 7A shows that there were no significant differences in renal tissue concentrations of HETEs (ω-hydroxylase AA metabolites) between the experimental FHL and FHH groups. Similarly, there were no significant differences in the renal tissue availability of biologically active epoxygenase metabolites of AA (expressed as the ratio of EETs to DHETEs) (Figure 7B). Untreated ACF FHL as well as ACF FHH rats displayed significantly lower ratios as compared with the sham-operated animals. The sEH inhibitor treatment markedly increased EETs/DHETEs ratios in ACF FHL rats and in ACF FHH rats to values that were even significantly higher than observed in sham-operated FHL and FHH rats.

Figure 7C demonstrates that LV tissue concentrations of HETEs did not significantly differ between the experimental groups of FHL and FHH rats. In addition, it shows that LV concentrations of HETEs were about 40% lower than observed in the kidney.

Figure 7D shows that untreated ACF FHL and ACF FHH did not exhibit lower EETs/DHETES ratios in the LV as compared with their sham-operated counterparts. However, the treatment with sEH inhibitor markedly increased EETs/DHETEs ratio in ACF FHL rats and in ACF FHH rats as compared with the sham-operated FHL and FHH rats.(more important I would be to say as compared with the untreated ACF FHL and ACF FHH rats) In addition, EETs/DHETEs ratio in the LV are was about three-fold higher than in the kidney.

**FIGURE 8:**
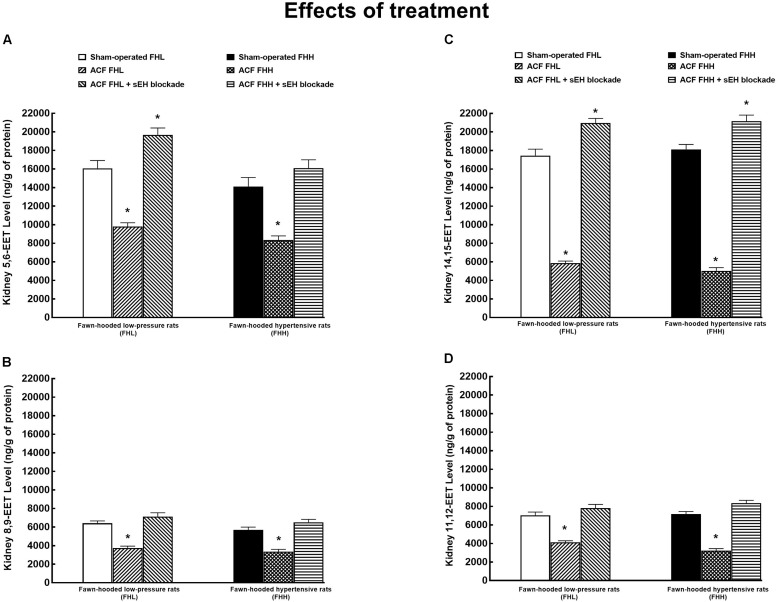
Renal tissue 5,6-epoxyeicosatrienoic acid (EET) **(A)**, 8,9-EET **(B)**, 14,15-EET **(C)**, and renal 11,12-EET **(D)** concentrations in FHL and hypertensive (FHH) rats. The data for the groups of either strain that were sham-operated or with ACF or ACF rats treated with a sEH inhibitor. ^∗^*P* < 0.05 compared with sham-operated counterparts.

Figure 8 shows the data on renal tissue availability of four biologically active epoxygenase products of AA. It is seen that ACF creation significantly decreased the levels of all EET regioisomers, to the same extent in FHL and FHH rats. The treatment with the sEH inhibitor restored these bioavailability values to levels observed in sham-operated animals of both strains. In addition, the data show that in FHL and in FHH rats 14,15-EET and 5,6-EET prevail among CYP-dependent epoxygenase metabolites of AA, which agrees with the results of previous studies indicating the critical importance of 14,15-EET in kidney tissue (Roman, 2002; Fleming, 2014; Capdevila et al., 2015; Jíchová et al., 2016; Imig, 2018).

**FIGURE 9:**
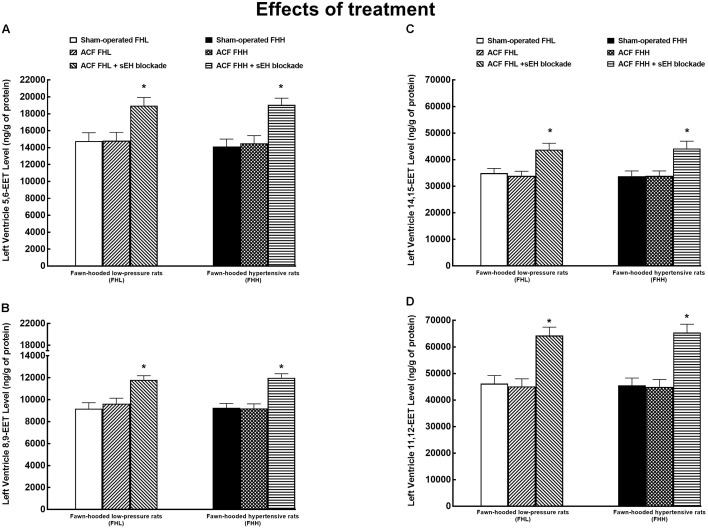
Left ventricle tissue 5,6-epoxyeicosatrienoic acid (EET) **(A)**, 8,9-EET **(B)**, 14,15-EET **(C)**, and renal 11,12-EET **(D)** concentrations in FHL and hypertensive (FHH) rats. The data for the groups of either strain that were sham-operated or with ACF or ACF rats treated with a sEH inhibitor. ^∗^*P* < 0.05 compared with sham-operated counterparts.

Figure 9 shows the data on LV tissue availability of four biologically active epoxygenase products of AA. It demonstrates that there were no significant differences in any of EET regioisomer concentrations between FHL and FHH rats in the LV. ACF creation did not alter LV concentrations of the EET regioisomers, similarly in FHL and FHH rats. The treatment with the sEH inhibitor increased LV levels of all EET regioisomers in FHL as well as in FHH rats to the same extent. In addition, the data show that in FHL and FHH rats 11,12-EET, and 14,15-EET prevail, which agrees with previous reports (Fleming, 2014; Jamieson et al., 2017). Moreover our data indicate that, quantitatively, increased concentrations of 11,12-EET and 14,15-EET are mostly responsible for the overall increment in LV tissue concentrations of biologically active epoxygenase products of AA.

**FIGURE 10:**
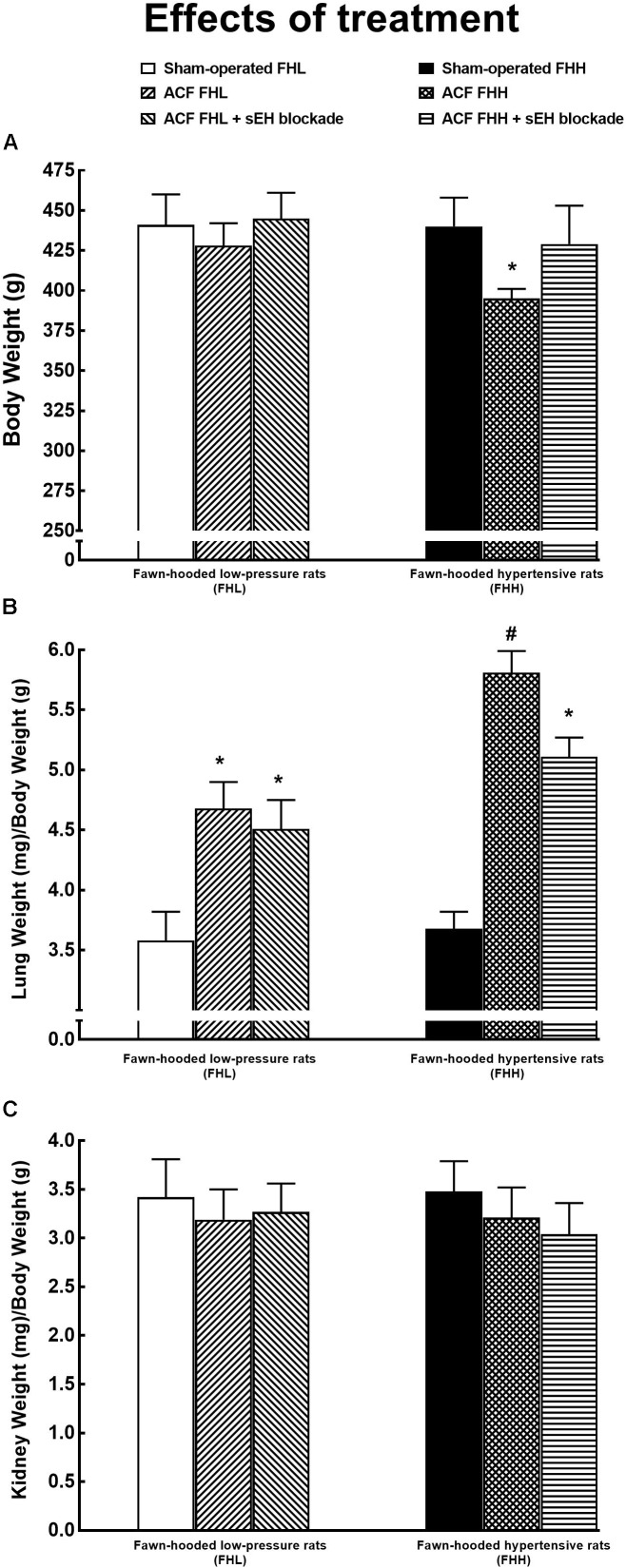
Body weight **(A)**, lung weight **(B)**, and kidney weight **(C)** normalized to body weight in FHL and hypertensive (FHH) rats. The data for the groups of either strain that were sham-operated or with ACF, or ACF rats treated with a sEH inhibitor. ^∗^*P* < 0.05 compared with the sham-operated counterparts. ^#^*P* < 0.05 significant difference from ACF FHH rats treated with sEH inhibitor.

As shown in Figure 10A, untreated ACF FHH rats exhibited significantly lower body weight as compared with all the other experimental groups.

Figure 10B shows that untreated ACF FHL as well as ACF FHH rats displayed significantly higher ratio of lung-to-body weight than their sham-operated counterparts, the increase was more pronounced in the ACF FHH group. These findings indicate the development of important lung congestion in ACF animals, particularly in untreated ACF FHH rats. The treatment with sEH inhibitor did not alter the ratio in ACF FHL but lowered it in ACF FHH rats. However, the ratio remained significantly higher than in sham-operated FHH rats.

There were no significant differences between experimental groups in the kidney weight when normalized to body weight (Figure 10C) and the liver weight (data not shown).

**FIGURE 11:**
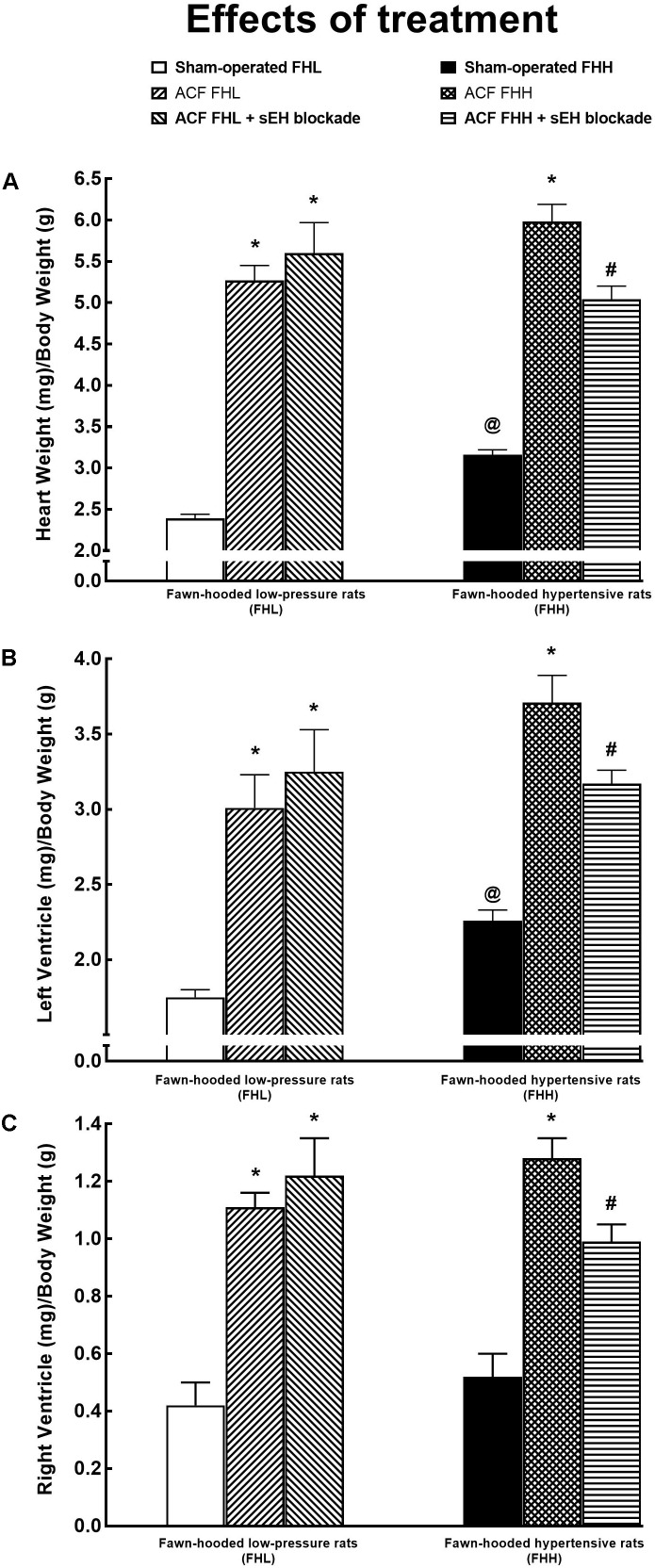
Whole heart weight **(A)**, left ventricle weight **(B)**, right ventricle weight **(C)** (all the values normalized to body weight) in sham-operated fawn-hooded low-pressure rats (FHL) with ACF, and ACF FHL rats treated with a sEH inhibitor, and in sham-operated fawn-hooded hypertensive (FHH) rats, untreated ACF FHH and ACF FHH treated with inhibitor of sEH. ^@^*P* < 0.05 for sham-operated FHH rats compared with sham-operated FHL rats. ^∗^*P* < 0.05 compared with sham-operated counterparts. ^#^*P* < 0.05 for ACF FHH rats treated with sEH inhibitor compared with untreated ACF FHH rats.

As shown in Figures 11A,B, sham-operated FFH rats exhibited significant cardiac hypertrophy [expressed as whole heart weight (HW) to body weight ratio], with marked left ventricle (LW) hypertrophy [expressed as LV weight (LVW) to body weight ratio] as compared with sham-operated FHL rats. Figure 11C shows that there were no significant differences in right ventricle (RV) weight (RVW) (expressed as RVW to body weight ratio) between sham-operated FHH and FHL rats.

Untreated ACF FHL and ACF FHH rats exhibited marked cardiac hypertrophy as compared with their sham-operated counterparts. The treatment with sEH inhibitor did not modify the development of cardiac hypertrophy in ACF FHL but, in contrast, attenuated it in ACF FHH rats (Figures 11A–C).

### Series 3: Effects of 2-Week Treatment With sEH Inhibitor on Basal Cardiac Function Parameters Assessed by Echocardiography and by Pressure-Volume Analysis

Table 1 summarizes the evaluation of cardiac function by echocardiography. The data confirms that sham-operated FHH displayed cardiac hypertrophy as compared with FHL rats and the induction of ACF caused marked cardiac hypertrophy, to the same extent in FHL and FHH, as soon as 6 weeks after ACF creation, The treatment with sEH inhibitor did not alter the degree of cardiac hypertrophy, similarly in sham-operated FHH or ACF FHL rats and ACF FHH rats. Untreated ACF FHL as well as ACF FHH rats showed increased stroke volume and cardiac output (dependent on the presence of the shunt), significant increases in LV and RV diameter, a decrease in the relative LV wall thickness (indicating the development of eccentric hypertrophy) and a significant decrease in LV fractional shortening (indication of LV systolic dysfunction) as compared with sham-operated FHL and FHH rats. The treatment with sEH inhibitor did not change any of these parameters, similarly in sham-operated FHL and FHH rats, and in ACF FHL and ACF FHH rats.

**Table 1:** Echocardiography at week 6 after induction of the aorto-caval fistula and after 2 weeks of treatment with the soluble epoxide hydrolase inhibitor.

	Group							
	
Parameter	FHL	FHL	ACF FHL	ACF FHL	FHH	FHH	ACF FHH	ACF FHH
	+	+	+	+	+	+	+	+
	water	sEHi	water	sEHi	water	sEHi	water	sEHi
Heart weight (mg)/Body weight (g)	2.78 ± 0.09	2.84 ± 0.08	4.99 ± 0.21*	5.12 ± 0.26*	3.21 ± 0.08**^#^**	3.22 ± 0.09**^#^**	5.34 ± 0.36*	5.30 ± 0.32*
RV diastolic diameter (mm)	3.25 ± 0.24	3.41 ± 0.29	6.19 ± 0.27*	6.24 ± 0.26*	3.31 ± 0.22	3.36 ± 0.27	6.21 ± 0.24*	6.20 ± 0.28*
LV diastolic diameter (mm)	6.19 ± 0.43	6.16 ± 0.47	11.02 ± 0.39*	10.99 ± 0.41*	6.17 ± 0.31	6.22 ± 0.33	10.87 ± 0.39*	11.04 ± 0.48*
LV systolic diameter (mm)	3.14 ± 0.38	3.17 ± 0.34	7.14 ± 0.38*	7.08 ± 0.34*	3.26 ± 0.21	3.27 ± 0.23	7.06 ± 0.24*	7.11 ± 0.27*
LV posterior wall thickness in diastole (mm)	2.36 ± 0.31	2.31 ± 0.29	2.27 ± 0.31	2.26 ± 0.29	2.96 ± 0.11	2.98 ± 0.10	2.28 ± 0.13	2.27 ± 0.13
Interventricular septum thickness (mm)	2.25 ± 0.18	2.23 ± 0.17	2.17 ± 0.17	2.18 ± 0.09	2.61 ± 0.17	2.63 ± 0.18	2.16 ± 0.18	2.17 ± 0.17
LV relative wall thickness (mm)	0.71 ± 0.05	0.69 ± 0.04	0.41 ± 0.03*	0.39 ± 0.04*	0.78 ± 0.04	0.79 ± 0.04	0.40 ± 0.03*	0.39 ± 0.03*
LV fractional shortening (%)	63 ± 3	61 ± 3	47 ± 2*	46 ± 2*	61 ± 3	62 ± 3	48 ± 2*	46 ± 3*
Stroke volume (μl)	206 ± 9	211 ± 8	556 ± 2*	562 ± 31*	209 ± 11	207 ± 9	573 ± 29*	569 ± 28*
Cardiac output (ml.min^1^)	108 ± 9	111 ± 12	308 ± 14*	318 ± 13*	112 ± 10	111 ± 9	321 ± 18*	322 ± 19*


*Values are means ± SEM. RV, right ventricle; LV, left ventricle; FHL, fawn-hooded low-pressure rats; FHH, fawn-hooded hypertensive rats; ACF, aorto-caval fistula; sEHi, soluble epoxide hydrolase inhibitor. ^∗^*P* < 0.05 compared with sham-operated counterparts. ^#^*P* < 0.05 FHH vs. FHL.*

Table 2 summarizes the assessment of cardiac function by LV pressure-volume analysis. Sham-operated FHH displayed only higher LV peak pressure as compared with sham-operated FHL rats (confirming a slight hypertension in FHH rats). With regard to all the other parameters there were no significant differences between sham-operated FHL and sham-operated FHH rats (indicating that sham-operated FHH rats do not exhibit any substantial impairment of basal cardiac function) and the treatment with sEH inhibitor did not change any parameter in sham-operated FHL and FHH rats.

**Table 2:** Invasive hemodynamics at week 6 after induction of aorto-caval fistula and after 2 weeks of treatment with soluble epoxide hydrolase inhibitor.

	Group							
	
Parameter	FHL	FHL	ACF FHL	ACF FHL	FHH	FHH	ACF FHH	ACF FHH
	+	+	+	+	+	+	+	+
	water	sEHi	water	sEHi	water	sEHi	water	sEHi
Heart rate (s^-1^)	435 ± 19	432 ± 21	419 ± 27	4271 ± 22	416 ± 29	418 ± 31	405 ± 29	409 ± 27
LV peak pressure (mmHg)	137 ± 4	139 ± 4	112 ± 4*	114 ± 4*	149 ± 4#	150 ± 3#	126 ± 3*#	127 ± 3*#
LV end-diastolic pressure (mmHg)	1.9 ± 0.5	1.9 ± 0.4	8.6 ± 0.8*	8.9 ± 0.7*	1.7 ± 0.6	1.8 ± 0.4	8.7 ± 0.7*	9.0 ± 0.9*
LV end-diastolic volume (ml)	0.29 ± 0.09	0.30 ± 0.08	0.99 ± 0.06*	1.04 ± 0.10*	0.31 ± 0.06	0.32 ± 0.09	1.08 ± 0.11*	1.06 ± 0.09*
+(dP/dt)_max_ (mmHg.s^-1^)	10972 ± 986	10732 ± 896	10742 ± 886	10963 ± 971	11563 ± 1001	11042 ± 952	11036 ± 896	11152 ± 992
-(dP/dt)_max_ (mmHg.s^-1^)	-9156 ± 832	-9063 ± 776	-9192 ± 874	-9098 ± 836	-9272 ± 914	-9154 ± 857	-9087 ± 798	-9108 ± 783
ESPVR_max_ (mmHg.ml^-1^)	286 ± 39	256 ± 33	57 ± 17*	61 ± 13*	261 ± 37	256 ± 36	54 ± 13*	58 ± 15*
EDPVR_max_ (mmHg.ml^-1^)	43 ± 9	39 ± 7	14 ± 4*	15 ± 4*	42 ± 6	40 ± 6	13 ± 4*	15 ± 4*
LV relaxation constant, tau (ms)	13 ± 2	14 ± 3	27 ± 4*	28 ± 3*	11 ± 3	12 ± 3	28 ± 3*	29 ± 3*


*Values are means ± SEM. LV, left ventricle; +(dP/dt)_*max*_, maximum rates of pressure rise; -(dP/dt)_*min*_, maximum rates of pressure fall; ESPVR_*max*_, maximum end-systolic pressure-volume relationship; EDPVR_*max*_, maximum end-diastolic pressure-volume relationship; FHL, fawn-hooded low-pressure rats; FHH, fawn-hooded hypertensive rats; ACF, aorto-caval fistula; sEHi, soluble epoxide hydrolase inhibitor. ^∗^*P* < 0.05 compared with sham-operated counterparts. ^#^*P* < 0.05 FHH vs. FHL.*

Untreated ACF FHL as well as ACF FHH rats showed markedly higher LV end-diastolic pressure and LV end-diastolic volume as compared with their sham-operated counterparts which indicated a significant degree of CHF in the two former groups. There were no significant differences in the maximum rate of pressure rise [+(dP/dt)_max_] and maximum rate of pressure fall [-(dP/dt)_max_] between the experimental groups. Untreated ACF FHL as well as ACF FHH rats showed markedly lower end-systolic pressure-volume relationship as compared with sham-operated FHL and sham-operated FHH rats, which indicated impairment of myocardial contractility in ACF animals. Untreated ACF FHL and ACF FHH rats also showed lower end-diastolic pressure volume relationship compared with their sham-operated counterparts, which indicated enhanced LV compliance resulting from the chamber eccentric remodeling. There were no significant differences in any measured parameter between ACF FHL and ACF FHH rats, and the treatment with sEH inhibitor did not significantly alter this situation, similarly in ACF FHL and ACF FHH rats.

## Discussion

Our interest in this study focused on the differences in the course of ACF-induced CHF in FHL versus FHH rats: first, we found that the latter animals exhibited substantially lower survival rate. It is emphasized that at the stage when ACF was induced, FHH rats showed only minimally increased albuminuria as compared with FHL rats. It will be noticed that while in adult FHH rats blood pressure (BP) is significantly higher than in FHL rats, at the age of 3–6 months FHHs are only slightly hypertensive. This was shown by our earlier radiotelemetry BP measurements: at the respective ages they reached mean BP levels of 138 and 148 mmHg, (Doleželová et al., 2016).

During the present follow-up study albuminuria increased to a greater extent in ACF FHH than in ACF FHL rats; at the week 10 (i.e., 14 weeks after creation of ACF) it was almost 30-fold higher and this was so despite the fact that ACF FHH rats with the most prominent albuminuria died earlier. In addition, our results show that urinary angiotensinogen excretion, a recognized index of the dynamics of intrarenal RAS activity (Kobori et al., 2003; Kobori and Urushihara, 2013) increased considerably more (almost 90-fold) in ACF FHH than in ACF FHL rats, indicating its much greater activation in the former. Furthermore, it was seen that ACF FHL rats as well as ACF FHH rats displayed reduced renal tissue availability of biologically active epoxygenase metabolites of AA, assessed as the ratio of EETs to DHETES (approximately 60% reduction as compared with sham-operated animals). In contrast, we found that myocardial tissue availability of biologically active epoxygenase metabolites of AA in ACF FHL rats or ACF FHH rats was not reduced as compared with their sham-operated counterparts. Broadly speaking, sham-operated FHH rats showed only slight BP increase (estimated from LV peak pressure) and only moderate cardiac hypertrophy as compared with sham-operated FHL rats whereas all the other indices of cardiac function as well as the response to sEH inhibitor were not altered. Creation of ACF resulted in similar responses in cardiac structure and function in FHL and FHH rats. Six weeks before ACF FHH rats began definitely to die, untreated ACF FHL as well as untreated ACF FHH rats developed marked eccentric LV remodeling and cardiac hypertrophy linked to increased cardiac output, the result of blood recirculation via the fistula. Even though the load-dependent measures of LV contractility [+(dP/dt)_max_] were at this stage not impaired, the suppression of the end-systolic pressure-volume slope and decreased LV fractional shortening imply significant impairment of systolic function. In addition, ACF FHL rats as well as ACF FHH rats also showed a suppression of the end-diastolic pressure-volume slope and prolongation of the LV relaxation, indicating impairment of early diastolic function. These findings are in accordance with the results of previous studies in this volume overload model of CHF (Brower et al., 1996; Oliver-Dussault et al., 2010; Červenka et al., 2015). However, the most important are our findings that there were no significant differences between ACF FHL rats and ACF FHH rats and that the treatment with sEH inhibitor, which significantly increased LV tissue availability of EETs, did not improve cardiac morphology and cardiac performance in ACF FHH rats, despite the fact that it dramatically improved the survival rate. These findings suggest that beneficial effects on BP or on the LV dysfunction are probably not the mechanism(s) responsible for the improvement of survival rate in ACF FHH rats treated with sEH inhibitor. In this context it is important to recognize that one important limitation of our present study is that the results provide only one time-point information on BP and cardiac function. It is still possible that in the advanced phase of CHF, alterations in cardiac function and/or BP might influence the outcome in ACF FHH rats. To achieve more meaningful comparative data, comprehensive long term telemetric studies are required that would evaluate BP and LV function. Unfortunately, technical difficulties, and relatively short durability of implantable telemetric probes limit at present the feasibility of such prolonged experiments.

Nevertheless, despite this limitation and after considering all our present data, we can conclude that even moderate kidney damage dramatically worsens the course of ACF-induced CHF in FHH rats. This further strengthens the notion on the critical importance of the heart and kidney interaction in the pathophysiology of progression of CHF. The two organs interact in a complex bidirectional mode, which can critical influence the outcome in patients with CHF (Braam et al., 2014; Schefold et al., 2016; Mullens et al., 2017; Arrigo et al., 2018; House, 2018).

The most important set of findings is that chronic pharmacological inhibition of sEH substantially improved survival rate and prevented increases in albuminuria in ACF FHL as well as ACF FHH rats. The beneficial effects of the treatment, particularly on the survival rate, were more pronounced in ACF FHH rats. In addition, our results show that, in accordance with previous findings (Oliver-Dussault et al., 2010; Melenovsky et al., 2012, 2018; Červenka et al., 2015, 2016; Kala et al., 2018), after creation of ACF the animals displayed signs of pronounced cardiac hypertrophy, involving both ventricles. Remarkably, this was associated with distinct lung congestion, indicating LV failure without signs of RV failure (no liver congestion). However, the signs of LV failure were more pronounced in ACF FHH rats and the treatment with sEH inhibitor attenuated cardiac hypertrophy and lung congestion in the hypertensive strain only, which further indicates that beneficial actions of the treatment are here more prominent. They were also associated with increases in intrarenal EETs availability; notably, *c-*AUCB-treated rats, both ACF FHL and ACF FHH strains, showed higher EETs/DHETEs ratio than found in their sham-operated counterparts. These findings accord with our recent report that in rats after 5/6 renal mass reduction (5/6 NX), a commonly used model of CKD, specific renoprotective effects (unrelated to the RAS blockade) are indeed associated with normalization of the intrarenal EETs availability (Čertíková Chábová et al., 2018).

In this context we have to acknowledge another important limitation of our present study: it did not evaluate the effects of chronic sEH inhibition on the development of hypertension and CKD in sham-operated FHH rats. In our previous study, we found (Doleželová et al., 2016) that renal concentrations of EETs were similar in young FHL and FHH rats, but in the latter renal EETs concentrations progressively decreased with age. This is important because of the known organ-protective actions of EETs (Elmarakby, 2012; Fan and Roman, 2017; Imig, 2018). Moreover, renal EETs deficiency was shown to significantly contribute to the pathophysiology of hypertension and renal damage in several models (Honetschlagerova et al., 2011; Elmarakby, 2012; Kopkan et al., 2012; Fan and Roman, 2017; Imig, 2018). Therefore, one can assume that progressively decreasing renal tissue EETs could contribute to the development of CKD in FHH rats and chronic pharmacologic sEH inhibition should attenuate it. Future long term, appropriately focused studies should address this issue, however, the present study (with a different focus) provides a necessary background for such studies.

On the other hand, our results show that intrarenal and myocardial activity of CYP-dependent ω-hydroxylase products (HETEs) was not significantly altered in ACF FHL or ACF FHH rats, and was not modified by chronic pharmacological sEH inhibition. These findings are important because increased activity of CYP-dependent ω-hydroxylase pathway of AA metabolism and increased production/action of HETEs (mainly 20-HETE) is proposed to substantially contribute to the pathophysiology of cardio-renal diseases (Jamieson et al., 2017; Rocic and Schwartzman, 2018). Our findings do not support this view, at least with the rat strain and experimental model used here. On the contrary, the results speak against the role of renal and myocardial alterations of the CYP-dependent ω-hydroxylase pathway of AA in the progression of CHF in ACF FHH rats.

In addition, our results show that, when assessed 14 weeks after ACF creation, ACF FHL and ACF FHH rats exhibited similar striking activation of both systemic and intrarenal vasoconstrictor/sodium retaining axis of the RAS, as indicated by elevation of plasma and kidney ANG II levels. However, it is striking that in ACF FHH rats urinary angiotensinogen excretion was dramatically higher (about 1000-fold) than observed in the ACF FHL strain. This suggests that the course of ACF-induced CHF in FHH rats is characterized by activation of intrarenal RAS that is more pronounced than observed in animals that are resistant to renal damage and development of CKD. We cannot provide satisfactory explanation why urinary angiotensinogen excretion and not renal tissue ANG II was the distinguishing feature between ACF FHH and ACF FHL rats. However, one should consider that ANG II levels were measured at the one time-point only and despite all precautions the very procedure of tissue sampling can alter the results (Husková et al., 2006a,b). On the other hand, urinary angiotensinogen excretion was performed repeatedly throughout the study and despite some technical limitations it allegedly presents a reliable marker for non-invasive evaluation of intrarenal RAS activity (Kobori et al., 2003; Kobori and Urushihara, 2013). At variance with the present observations, in a recent study of 5/6 NX rats we showed a high correlation between renal ANG II concentrations and urinary angiotensinogen excretion (Sedláková et al., 2017; Čertíková Chábová et al., 2018). Nevertheless, regardless of the reason(s) for the discrepancy between urinary angiotensinogen excretion and renal ANG II concentrations, an assessment of both parameters clearly shows that the intrarenal RAS system was activated, and that sEH inhibition did not alter this activation, quite, similarly so in ACF FHL and ACF FHH rats. This indicates that the beneficial effects of chronic sEH inhibition are not related to alterations of the vasoconstrictor/sodium retaining axis of the RAS, which further supports the notion that CHF is not simply a hemodynamic disorder. Apparently, compensatory activation of systemic and tissue neurohormonal systems, perhaps also outside the heart and the kidney, plays an important role in the progression of CHF and can in the long-term can adversely affect the outcome of CHF, probably due to inappropriate activation of the RAS and/or insufficient activation of the CYP epoxygenase enzymatic pathway (Dube and Weber, 2011; Hartupee and Mann, 2017; Packer and McMurray, 2017).

## Conclusion

We believe that the results of our present study provide further evidence that an association of even mild form of CKD has a strikingly negative impact on the course of ACF-induced CHF and that the reduced renal availability of EETs plays an important role. We found that sEH inhibitor treatment normalized renal availability of EETs and improved the long-term survival rate, without altering RAS activity. The results provide the rationale for attempts to increase the generation of EETs, possibly by reducing their degradation by sEH, as a new therapeutic approach for the treatment of CHF, particularly of its forms associated with CKD.

## Author Contributions

ŠV, LK, JS, EK-J, JI, MT, VM, and LČ primarily conceived and designed the study, analyzed, and interpreted the data, and wrote the manuscript. ŠV, LK, and LČ performed all surgical procedures in this study. SK and ZH performed the biochemical analyses. BH designed and synthesized the sEH inhibitor. MT and VM performed studies evaluating cardiac function. All authors were involved in the final analysis and interpretation of the data and contributed to the intellectual content and editing of the manuscript, and approved its final version.

## Conflict of Interest Statement

The authors declare that the research was conducted in the absence of any commercial or financial relationships that could be construed as a potential conflict of interest.
